# Supplementary material for: Keeping Sane in a Changing Climate: Assessing Psychologists’ Preparedness, Exposure to Climate-Health Impacts, Willingness to Act on Climate Change, and Barriers to Effective Action
Source: Int J Environ Res Public Health. 2024 Feb 13;21(2):218. doi: 10.3390/ijerph21020218 (PMC10888424; doi:10.3390/ijerph21020218)
Supplement: Supplementary file 1 [file ijerph-21-00218-s001.zip › ijerph-2798677-supplementary.pdf]

Keeping sane on a changing climate: assessing Australian Psychologists' preparedness, exposure to

The next questions are demographic questions that will help us understand who you are.

1. What type of registration do you hold with AHPRA?

- ◯ Provisional registration
- ◯ General Registration without endorsements
- ◯ General with endorsements
- ◯ Non-practising registration
- ◯ I don't hold a registration as psychologist with AHPRA

2. Which endorsement do you hold?

- ◯ Clinical psychology
- ◯ Counseling psychology
- ◯ Forensic psychology
- ◯ Clinical neuropsychology
- ◯ Organisational psychology
- ◯ Sport and exercise psychology
- ◯ Educational and developmental psychology
- ◯ Health psychology
- ◯ Community psychology

3. Have you ever been registered/ done your educational training (6+ months) overseas?

- ○ No
- ○ Yes

4. How long have you been a registered psychologist (including provisional registration) in Australia?

- ○ Less than 5 years
- ○ 5 to 10 years
- ○ 10 to 15 years
- ○ More than 15 years
- ○ Prefer not to say

5. How old are you?

- ○ 18-24 years old
- ○ 25-34 years old
- ○ 35-44 years old
- ○ 45-54 years old
- ○ 55-64 years old
- ○ 65 + years old
- ○ Prefer not to say

6. Which most closely describes your gender?

- ○ Woman
- ○ Man
- ○ Transgender woman/ man
- ○ Non-binary
- ○ Agender
- ○ I prefer not to say

7. What is your postcode?

____

8. Considering your position as a psychologist/provisional psychologist, do you work (please mark all that applies to you)

- ☐ In direct client contact (eg. providing psychological interventions)
- ☐ In an academic/teaching role
- ☐ In a research role
- ☐ In leadership /senior roles without client contact
- ☐ In policy-making (eg. government)

9. Considering your position as a psychologist/provisional, what is your main work setting?

- ○ Private practice
- ○ School
- ○ Hospitals
- ○ Community mental health services
- ○ Private Organisations other than clinical private practice
- ○ Government
- ○ Other ____

10 Considering your position at work, to which degree are you in a decision-making position in your workplace?

- ○ Not at all
- ○ Somewhat, mostly decide on things that affect me and my clients directly (eg. calendar)
- ○ I am responsible for a team within a bigger organisation
- ○ I am fully responsible for decisions in my organisation (eg. I'm the owner)

We have finished with the demographic questions, thank you. Now we will ask questions about climate change.

11 How interested are you in the topic of climate change?

- ○ Not at all interested
- ○ Not very interested
- ○ Somewhat interested
- ○ Very interested

12 How important is the issue of climate change to you personally?

- ◯ Not at all important
- ◯ Somewhat important
- ◯ Very important
- ◯ Extremely important

13 When thinking about climate change, do you feel any of the following emotions ? (tick all that applies)

- [ ] Frustration
- [ ] Worry
- [ ] Sadness
- [ ] Outrage
- [ ] Overwhelm
- [ ] Despair
- [ ] Disbelief
- [ ] Fear
- [ ] Confusion
- [ ] Grief
- [ ] Anxiety
- [ ] Guilt
- [ ] Joy
- [ ] I don't have an emotional reaction to climate change

14. And to what extent to you experience those emotions you have selected?

	A little	Moderately	a lot
Frustration	○	○	○
Worry	○	○	○
Sadness	○	○	○
Outrage	○	○	○
Fear	○	○	○
Overwhelm	○	○	○
Despair	○	○	○
Disbelief	○	○	○
Confusion	○	○	○
Grief	○	○	○
Anxiety	○	○	○
Guilt	○	○	○
Joy	○	○	○

15 How interested do you think your peers are in climate change?

- ◯ Not at all interested
- ◯ Not very interested
- ◯ Somewhat interested
- ◯ Very interested
- ◯ Unsure
- ◯ Prefer not to say

16. How often does the subject of climate change arise in your workplace?

- ○ Never
- ○ Sometimes
- ○ Occasionally
- ○ Quite often
- ○ Very often

17. In the past 12 months, has your workplace been affected by climate change and extreme weather events? (Select all that apply)

- ☐ No
- ☐ I don’t know
- ☐ Bushfires
- ☐ Bushfire smoke
- ☐ Cyclones
- ☐ Extreme heat or heatwaves
- ☐ Flooding
- ☐ Storms
- ☐ Droughts
- ☐ other ____

18. How has your workplace been affected by extreme weather, or natural disaster? (Select all that apply)

- ☐ Demand for services increased
- ☐ Extra staff needed
- ☐ Staff transport interrupted
- ☐ Staff health affected
- ☐ Staff absent from work
- ☐ Client access to services was affected
- ☐ Premises/assets destroyed or damaged
- ☐ We had to relocate
- ☐ Power outage
- ☐ None of these
- ☐ Don’t know
- ☐ Other ____

We will now ask about climate-related health impacts. Please remember this is not an evaluation of your knowledge

19 Do you agree that climate change affects human health?

- Definitely not
- Probably not
- Might or might not
- Probably yes
- Definitely yes

20 How well informed do you feel about the potential human health impacts of climate change?

- Not at all informed
- Not very informed
- Somewhat informed
- Well informed
- Very well informed

20. Have your clients/patients' health been affected by climate change?

- Yes
- No
- I am not sure
- I don't work in direct contact with clients

21. In which way did climate change affected your client/patients health?

____

____

____

____

____

22. If your clients/patients/communities had experienced mental health distress (clinical and non-clinical) due to climate change, what are the most common presentations:

- ☐ anxiety directly related to severe weather events
- ☐ anxiety about the future and the loss of biodiversity (eco-anxiety)
- ☐ depression directly related to severe weather events
- ☐ depression about the future
- ☐ depression about the the loss of biodiversity (solastagia)
- ☐ PTSD
- ☐ I am yet to see a presentation related to climate change
- ☐ other ____
- ☐ I am not sure/ I don't know

23. To what extent do you feel prepared to help your client/patient with health issues related to climate change?

- ◯ Not at all prepared
- ◯ Not very prepared
- ◯ Somewhat prepared
- ◯ Very prepared
- ◯ Unsure
- ◯ Prefer not to say

24. Have you ever had any training in climate change and its impact on health?

- ◯ Not at all
- ◯ Not really, just some readings on my own
- ◯ Somewhat, nothing longer than 1 hour
- ◯ Yes, I attended a training longer than 1 hour
- ◯ Unsure
- ◯ Prefer not to say

25 To what extent do you agree with the following statements?

	Strongly disagree	Somewhat disagree	Neither agree nor disagree	Somewhat agree	Strongly agree
Climate change is a serious problem that we need to take immediate action on	○	○	○	○	○
The public need to be better informed about the link between climate change and health	○	○	○	○	○
Health professionals are well informed about climate change and its impact on health	○	○	○	○	○

26. Is tackling climate change already happening or is planned for in your workplace?

- ◯ Yes
- ◯ No
- ◯ Don't know

27. How is your workplace tackling climate change?

- ☐ Reducing emissions
- ☐ Advocating for policy to address climate change at the federal, state and local levels
- ☐ Diverting from fossil fuels (eg. changing financial institutions, promoting active transportation, etc.)
- ☐ Providing training for all staff on climate change
- ☐ Other ____

28 What do you think your workplace could be doing to tackle climate change?

- ☐ Take actions to reduce emissions
- ☐ Advocate for policy to address climate change at the federal, state, and local levels
- ☐ Divest from fossil fuels
- ☐ Provide training for all staff on climate change
- ☐ Support the population to cope with climate change
- ☐ Provide information/ help the public to change behaviour towards acting on climate change
- ☐ Don't know
- ☐ I don’t believe it is my organisation’s role to tackle climate change
- ☐ Other ____

29 What barriers does your workplace face in tackling climate change?

- [ ] There are financial constraints
- [ ] There is no support from leaders or management in my organisation
- [ ] A lack of political will
- [ ] My organisation doesn’t have time to work on climate change
- [ ] Climate change is not a priority in my organisation
- [ ] It’s not clear how my organisation can tackle climate change
- [ ] Don't know
- [ ] Other ______________________________

30. Do you or others in your workplace currently communicate with patients, clients, or the community about the health impacts of climate change? (Select all that apply)

- [ ] Yes - I do
- [ ] Yes - others do
- [ ] Don't know if others do
- [ ] No
- [ ] Other ______________________________

31. How comfortable are you with doing these things in your professional practice?

	Not at all comfortable	Not particularly comfortable	Slightly uncomfortable	Neither comfortable nor uncomfortable	Somewhat comfortable	Very comfortable	Not applicable
Communicate the health impacts of climate change to your patients/clients	○	○	○	○	○	○	○
Communicate the way your patients/clients can protect themselves from the health effects of climate change	○	○	○	○	○	○	○
Communicate to your patients/clients the actions and day to day changes people in the broader community can make to prevent climate change getting worse	○	○	○	○	○	○	○

32. What, if anything, prevents you from taking these actions? (select all that applies)

- ☐ I do not feel well informed enough about the health impacts of climate change
- ☐ I do not feel it is my responsibility to communicate the health impacts of climate change
- ☐ My organisation would not support me to communicate the health impacts of climate change to my patients/clients
- ☐ I don’t believe it is ethical to discuss climate change unless the topic comes from my client/patient
- ☐ Climate change is not a priority in my practice/life
- ☐ Nothing prevents me, I already do these things
- ☐ Don't know
- ☐ Other ____

33. To what extent do you agree with the following statements?

	Strongly Disagree	Disagree	Neither agree nor disagree	Somewhat agree	Strongly agree
As a psychologist, I am sufficiently informed about the health impacts of climate change	○	○	○	○	○
I would like to know more about the health impacts of climate change	○	○	○	○	○
There are sufficient education and training opportunities available to inform me, and keep me updated, on the issue of climate change and public health	○	○	○	○	○
Climate change, its health impacts and mitigation opportunities should be covered in the undergraduate curriculum for health related professions	○	○	○	○	○

Climate change, its health impacts and mitigation opportunities should be part of ongoing professional training	○	○	○	○	○
Psychologists definitely have a role to play in informing the public about the health impacts of climate change	○	○	○	○	○
Psychologists have a responsibility to endure their practice is tackling climate change	○	○	○	○	○
If no significant changes are made to combat climate change, health services and health structures will face significant challenges due to increasing demand for services and increased risks to infrastructure	○	○	○	○	○

34. Are you familiar with any Psychology Professional Association/Society position statement on climate change?

- Not at all familiar
- Somewhat familiar
- Well familiar

35. Which Professional Association/Society position statement on climate change are you familiar with?

____

____

____

____

____

36 How likely would you be to do the following:

	Extremely unlikely	Somewhat unlikely	Neither likely nor unlikely	Somewhat likely	Extremely likely
Advocate for climate action in your local community to reduce the risks to the population's health	○	○	○	○	○
Advocate / make the decision for your organisation to develop a climate risk plan	○	○	○	○	○
Advocate/make the decision for your organisation to reduce its emissions	○	○	○	○	○

37. What, if anything, currently prevents you from taking these actions? (Select all that apply)

- [ ] I don’t feel informed/trained enough about the health impacts of climate change
- [ ] I don’t feel comfortable advocating for action on climate change
- [ ] I think it is an ethical infringement to advocate on climate change
- [ ] I don’t know how to advocate for action on climate change
- [ ] Climate change is not a priority to me
- [ ] It’s not clear how my organisation can tackle climate change
- [ ] There is no support from leaders/management in my organisation to act on climate change
- [ ] Don't know
- [ ] Other ____

That’s it, you have finished!! Well done! Thank you very much for taking the time to share your thoughts and opinions.

Just one last question: Would you like to register your email to participate in further research on this topic or to receive updates on the study findings?

- ○ Yes
- ○ No

Please provide an email so we can provide you with updates on the study findings.

____
